# Robot‐Assisted Cystoprostatectomy for Muscle‐Invasive Bladder Cancer in a Patient With a Prior Simultaneous Pancreas–Kidney Transplantation

**DOI:** 10.1155/crit/9534770

**Published:** 2026-02-06

**Authors:** Laetitia Lonca, Peter Beniac, Andrei Necsulescu, David Teixeira-Guerra, Bastien Parier, Jacques Irani, Sophie Ferlicot, Thomas Bessede, Renaud Snanoudj, Cedric Lebacle

**Affiliations:** ^1^ Urology Department, Bicêtre Hospital, Assistance Publique Hôpitaux de Paris, Paris-Saclay University, Le Kremlin-Bicêtre, France, universite-paris-saclay.fr; ^2^ Pathology Department, Bicêtre Hospital, Assistance Publique Hôpitaux de Paris, Paris-Saclay University, Le Kremlin-Bicêtre, France, universite-paris-saclay.fr; ^3^ Nephrology and Transplantation Department, Bicêtre Hospital, Assistance Publique Hôpitaux de Paris, Paris-Saclay University, Le Kremlin-Bicêtre, France, universite-paris-saclay.fr

## Abstract

**Introduction:**

Kidney transplant recipients are at increased risk of malignancy due to chronic immunosuppression. Although rare (≈0.3%), bladder cancer in this population tends to be aggressive and may present with atypical symptoms. We report a case of muscle‐invasive bladder cancer in a patient with prior simultaneous pancreas–kidney transplantation, successfully managed by fully intracorporeal robot‐assisted radical cystoprostatectomy and urinary diversion.

**Presentation:**

A 46‐year‐old male with a history of a simultaneous pancreas and kidney transplantation presented with persistent storage lower urinary tract symptoms. Cystoscopy revealed a necrotic, atypical lesion with associated inflammatory changes. Preoperative CT urography showed thickening of the bladder dome, with peripheral enhancement and a calcified component. The patient underwent robot‐assisted radical cystoprostatectomy with intracorporeal ileal conduit involving the transplanted kidney.

**Clinical Discussion:**

This rare case highlights the importance of thorough urological evaluation in transplant recipients presenting with atypical urinary symptoms.

**Conclusion:**

This case demonstrates the feasibility and safety of fully intracorporeal robot‐assisted cystoprostatectomy with urinary diversion in a patient with a simultaneous pancreas–kidney transplant.

## 1. Introduction

Immunosuppressive drugs are known to increase the risk of developing solid tumors (1). Furthermore, transplant recipients have more aggressive cancer and poorer prognostic outcomes compared to the general population (2). Incidence of urothelial carcinoma in renal chronic disease and transplant patients is approximatively three times the incidence of general population. This average is based on relative risks (RRs) reported in major registries: 1.4 [1.3–1.5] in the United States, 1.5 [1.4–1.7] in Europe, and 4.8 [3.6–4.2] in Australia and New Zealand (3). A recent Korean cohort confirmed a significantly increased risk of genitourinary cancers, particularly of the bladder and kidney, in older male transplant recipients (above 60 years) (4).

According to the French Urological Association′s transplantation committee (CTAFU), surgical management remains the standard of care for localized invasive bladder cancer without metastasis or lymph node involvement, in line with general population guidelines (5). This report follows the SCARE (Surgical CAse REport) 2023 guidelines for structured and transparent surgical case reporting (6). We report here a complex surgical case illustrating the feasibility of fully intracorporeal robot‐assisted cystoprostatectomy and urinary diversion in a patient with simultaneous pancreas–kidney transplantation.

## 2. Case Report

A 46‐year‐old man with a history of simultaneous pancreas and kidney transplantation in 2005 for insulin‐dependent diabetes mellitus presented with persistent storage lower urinary tract symptoms (LUTSs). He denied any recent trauma, urinary tract infection, or tobacco use. His past medical history included urethroplasty for a urethral stricture secondary to condylomatosis. Initial posttransplant follow‐up showed satisfactory pancreatic and renal allograft function. However, over time, a ureterohydronephrosis of the transplanted kidney developed due to ureterovesical anastomotic stricture, leading to impaired renal function.

**Figure 1:**
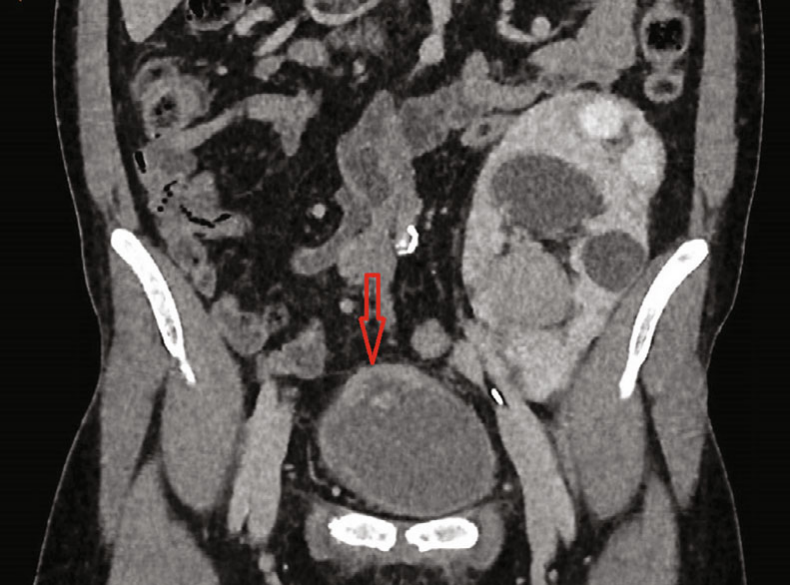
Lesion (4 × 4 × 2 cm) of the bladder dome (arrow). We can observe ureterohydronephrosis of the transplanted kidney.

In 2024, cystoscopy revealed a necrotic lesion at the bladder dome with surrounding inflammation. Given the proximity of the pancreatic graft and the suspicion of a fistulous tract, a CT urogram was performed. It showed bladder dome wall thickening with peripheral enhancement and a calcified component, adjacent to the pancreatic graft (Figure 1).

**Figure 2:**
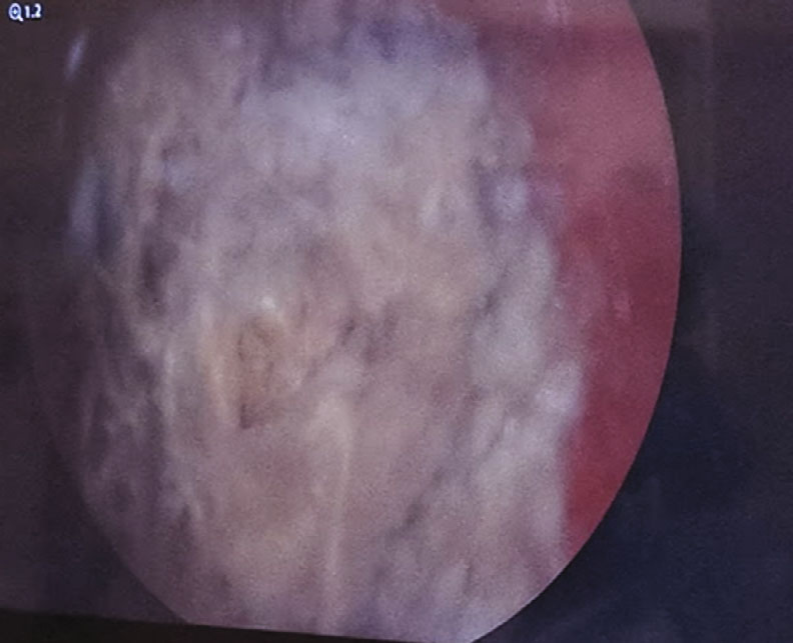
Endoscopic vision of principal bladder lesion during transurethral resection.

A transurethral resection (TUR) of the lesion and random biopsies of inflamed mucosa were performed. Simultaneously, retrograde ureteropyelography confirmed the ureterovesical stricture, and a ureteral stent was placed (Figure 2).

Histopathological analysis of the necrotic lesion revealed high‐grade urothelial carcinoma with squamous differentiation infiltrating the deep lamina propria, but without identified detrusor muscle in the specimen classified at least as pT1 (AJCC eighth edition). Random biopsies were also positive for high‐grade urothelial carcinoma. Staging with FDG‐PET scan showed no evidence of nodal or distant metastases. Following tumor board discussion, imaging was considered consistent with muscle‐invasive bladder cancer possibly invading the surrounding fat. Due to impaired renal function and the patient′s history of simultaneous pancreas–kidney transplantation, neoadjuvant chemotherapy was not indicated. Given the high risk associated with pelvic lymphadenectomy in the context of vascularized grafts along the iliac axis, a robot‐assisted radical cystoprostatectomy without lymphadenectomy was proposed as the most appropriate therapeutic option. After extensive preoperative counseling regarding the risks and options and given his age (46 years old), the patient opted for a neobladder reconstruction, fully aware of the possibility of intraoperative conversion to an ileal conduit due to the anatomical constraints posed by the pancreatic graft.

Immunosuppressive treatment of the patient consisted of everolimus 5 mg two times a day and prednisone 5 mg once a day. Everolimus was progressively replaced by tacrolimus. Tacrolimus was administered at the posology of 0.1 mg/kg in two doses daily until we reached 3 mg twice a day. Everolimus was fully stopped 10 days before surgery.

### 2.1. Surgical Technique

**Figure 3:**
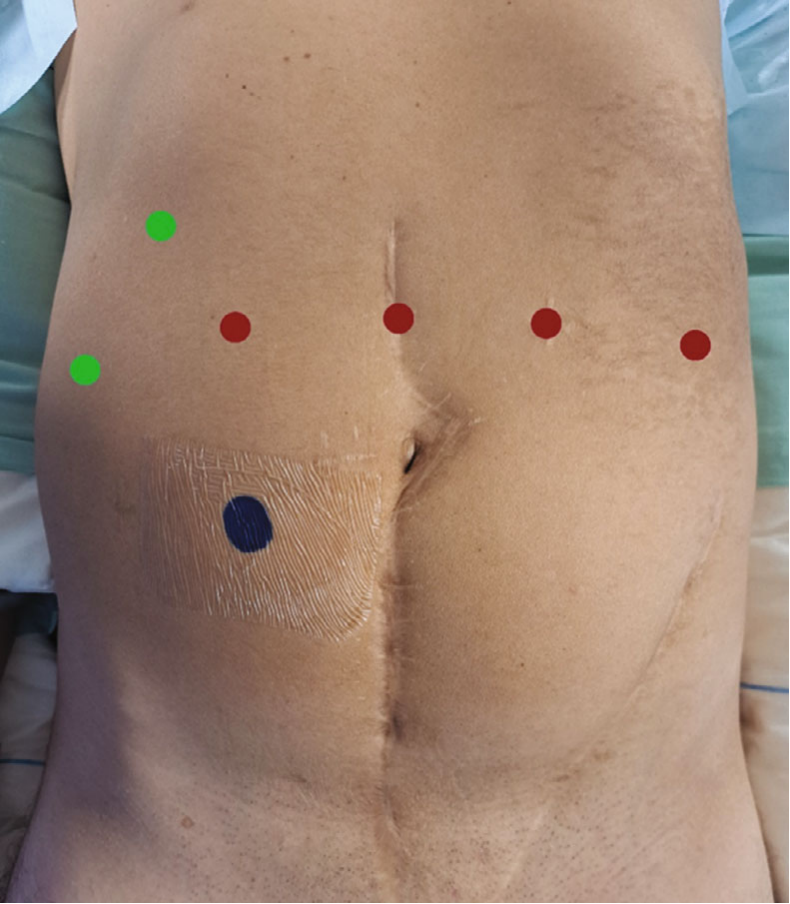
Trocar placement for robot‐assisted radical cystoprostatectomy and urinary diversion. Red dots indicate robotic trocars: one 12‐mm trocar in the left flank for the robotic stapler, one robotic trocar in the left iliac fossa, and one robotic trocar in the right lower quadrant. The central red dot above the umbilicus corresponds to the camera port. Green dots indicate assistant trocars: one 12‐mm trocar placed in the right hypochondrium used for open entry (Hasson technique) and assistant instrumentation and one AirSeal trocar in the right flank for insufflation and assistant instrumentation. The blue dot in the left iliac fossa represents the preoperatively marked site for the ileal conduit (stoma).

The patient was placed in a steep Trendelenburg position (20°). Trocar positioning during the whole intervention is illustrated in Figure 3. Red dots indicate robotic trocars: one 12‐mm trocar in the left flank for the robotic stapler, one 8‐mm robotic trocar in the left iliac fossa, and one 8‐mm robotic trocar in the right lower quadrant. The central red dot above the umbilicus corresponds to the 8‐mm camera port. Green dots indicate assistant trocars: one 12‐mm trocar placed in the right hypochondrium used for open entry (Hasson technique) and assistant instrumentation and one AirSeal trocar in the right flank for insufflation and assistant instrumentation. The blue dot in the left iliac fossa represents the preoperatively marked site for the ileal conduit (stoma) if needed.

The pancreatic graft was found intraoperatively to be densely adherent to the bladder dome. Careful dissection was necessary, including resection of a small portion of peripancreatic fat. Intraoperative frozen section analysis confirmed the absence of malignancy at the margin. The native ureters were identified, clipped near their bladder insertions, and transected. The peritoneum was opened at the rectovesical pouch, and dissection continued along the posterior and anterior mesorectal planes. The prostate was mobilized, and the posterior urethra was exposed. The bladder was removed en bloc with the prostate.

Despite initial attempts at neobladder reconstruction, the mesentery could not be mobilized sufficiently toward the urethra due to the fixed retroperitoneal position of the pancreatic graft. The decision was made to convert to an extended ileal conduit. A 40‐cm ileal segment was harvested, and the left‐sided ureter of the renal graft was transected from the bladder and anastomosed to the ileal conduit. A single‐J stent was inserted into the renal pelvis through the conduit. The ileal conduit was brought out in the right iliac fossa at the site marked preoperatively. The specimen was retrieved in an endobag through a short periumbilical laparotomy. A drain was placed adjacent to the pancreatic graft, and standard fascial and skin closure completed the procedure. The intervention lasted 9 h. Estimation of blood loss was 900 mL.

### 2.2. Investigations and Treatment

**Figure 4:**
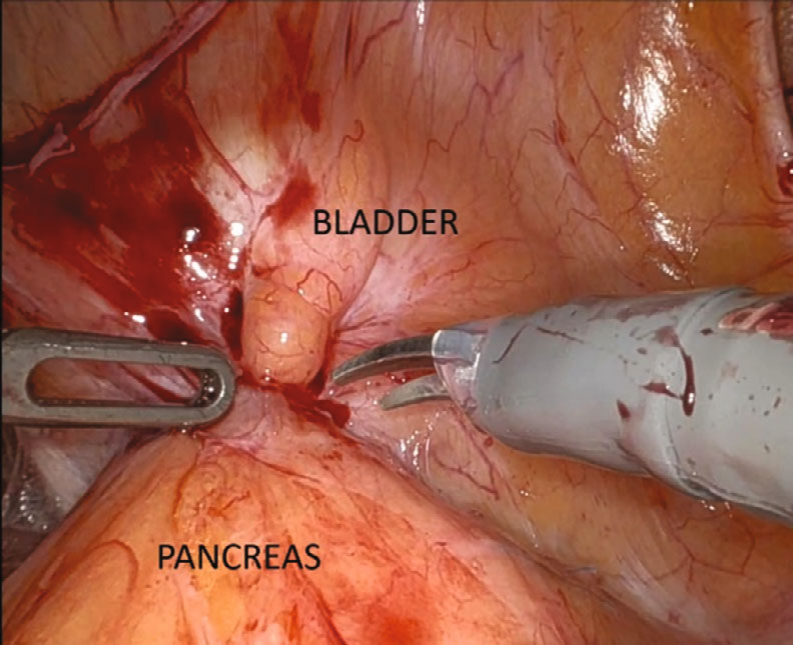
External view of the bladder lesion in close apposition to the pancreatic graft.

The patient underwent a robot‐assisted cystoprostatectomy with intracorporeal ileal conduit involving the renal graft. An intraoperative image captured the bladder lesion adherent to the pancreatic transplant (Figure 4).

#### 2.2.1. Histopathology

**Figure 5:**
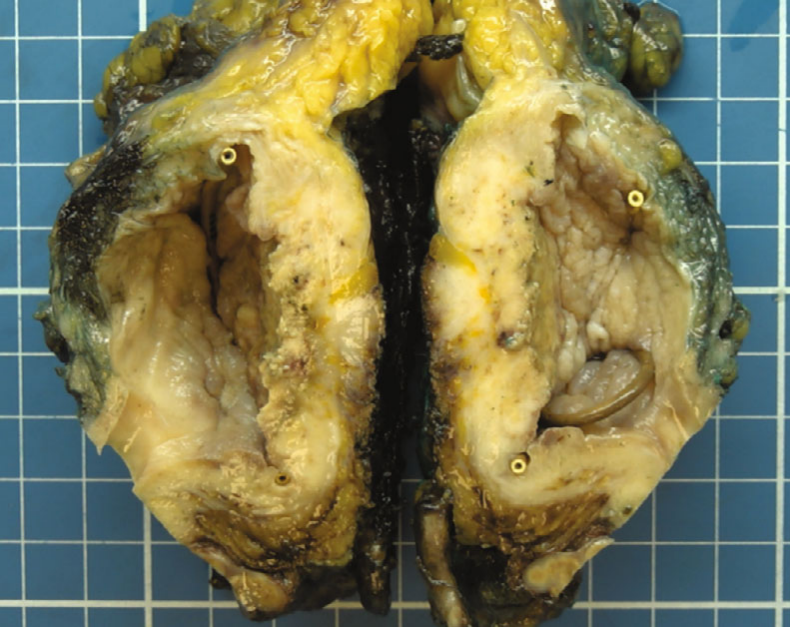
Gross pathology showing a 4‐cm necrotic and infiltrative lesion involving the bladder dome and posterior wall, extending into perivesical adipose tissue.

Macroscopic evaluation revealed an indurated lesion with a necrotic center, measuring 4 cm along its longest axis, located on the posterior bladder wall and dome, infiltrating down to the perivesical adipose tissue (Figure 5). Histopathological analysis confirmed a 4‐cm high‐grade urothelial carcinoma with predominant squamous differentiation, infiltrating the perivesical adipose tissue and demonstrating perineural invasion. Final pathological staging was pT3b Nx R0 (AJCC eighth edition).

#### 2.2.2. Postoperative Course and Follow‐Up

The immediate postoperative period was marked by several complications. Extensive intraoperative viscerolysis contributed to delayed return of bowel function. Renal function deteriorated following removal of the single‐J stent, with serum creatinine remaining elevated at 200 *μ*mol/L compared to a nadir of 132 *μ*mol/L. This required close follow‐up in collaboration with the nephrology team who managed the immunosuppressive therapy by adjusting tacrolimus based on trough levels. On Postoperative Day 15, a 6‐cm pelvic collection became infected, requiring percutaneous drainage and reinsertion of a single‐J stent. The patient was discharged on Postoperative Day 35 following completion of intravenous antibiotic therapy and immunosuppressive balance.

#### 2.2.3. Midterm Follow‐Up

At 3 months, the patient was readmitted for a subocclusive episode due to adhesions near the paraumbilical intestinal anastomosis, which resolved after 4 days of nasogastric decompression. The single‐J stent was changed at 3 months and definitively removed at 6 months, at which point renal function had stabilized (creatinine 168 *μ*mol/L). One month after stent removal, renal function remained stable, with a creatinine of 149 *μ*mol/L and an estimated glomerular filtration rate of 48 mL/min/1.73 m^2^ (CKD‐EPI).

## 3. Discussion

LUTSs are common after kidney transplantation. A recent meta‐analysis reported a prevalence of nonneurogenic LUTS ranging from 5.8% to 33.0% (7). Among these patients, urologic aftercare may detect bladder urothelial carcinoma at an early stage. It is essential to ensure prompt diagnosis and tailored management as immunosuppressed transplant recipients with cancer face worse outcomes than the general population (2). Urothelial malignancies are scarce but their RR is significantly increased, making them a notable source of morbidity and mortality in this population (8, 9). Current recommendations for the management of bladder cancer in transplant recipients largely mirror those for nontransplanted patients (5). Robotic surgery is increasingly reported in this setting. In 2020, a case from Tokyo described a robot‐assisted cystectomy with extracorporeal urinary diversion in a kidney transplant recipient (10). More recently, in 2025, Tillou et al. underwent the first full intracorporeal robotic neobladder in a kidney‐transplanted patient (11).

Our case was particularly challenging due to the anatomical consequences of the prior combined kidney–pancreas transplantation. Initially, a pancreatic fistula eroding the bladder wall was suspected, potentially contributing to a delay in diagnosis. Furthermore, we had intraoperative anatomical difficulties; the sacral position of the pancreas graft prevented adequate mesenteric descent for a neobladder. This ultimately required conversion to an ileal conduit. Moreover, immunosuppressive treatment was adjusted preoperatively. mTOR inhibitors have been shown to be an effective treatment modality in transplant recipients as both an antineoplastic therapy in the treatment of skin cancers; however, they have significant potential side effects including reduced wound healing (12). Despite these challenges, transplantation remains beneficial: A recent Korean cohort found that the adjusted hazard ratio for bladder cancer survival was 0.29 in transplant recipients compared to dialysis patients, with landmark analyses at 2 and 5 years supporting long‐term benefit (13).

Optimal management of these patients requires a coordinated multidisciplinary approach, including nephrologists, urologists, anesthesiologists, and oncologists. In this case, adjuvant immunotherapy was discussed but ultimately not pursued. The patient remains under close surveillance. Emerging research into the role of immune mechanisms in bladder carcinogenesis may further refine risk stratification and therapeutic strategies in transplant populations (14).

## 4. Conclusion

There are currently no specific guidelines for the management of urothelial carcinoma in transplant recipients. However, existing recommendations tend to parallel those applied in the general population. This case underscores the surgical and perioperative complexity involved in treating invasive bladder cancer in patients with prior simultaneous pancreas–kidney transplantation and highlights the feasibility of a fully robotic approach in this challenging context.

## Conflicts of Interest

The authors declare no conflicts of interest.

## Funding

No funding was received for this manuscript.

## Data Availability

No new data were analyzed in this study. Data sharing is not applicable to this article.
